# Lubricin in synovial fluid of mild and severe temporomandibular joint internal derangements

**DOI:** 10.4317/medoral.21145

**Published:** 2016-10-01

**Authors:** Rosalia Leonardi, Rosario E. Perrotta, Luis-Eduardo Almeida, Carla Loreto, Giuseppe Musumeci

**Affiliations:** 1 Department of Orthodontics and Orofacial Pain, University of Catania, Catania, Italy; 2 Department of Medical and Surgical Science. Section of Plastic Surgery. University of Catania, Catania, Italy; 3 Department of Surgical Sciences- Oral surgery, Marquette University School of Dentistry, Milwuakee, Wisconsin, USA; 4 Department of Bio-Medical Sciences, Anatomy Section, University of Catania, Catania, Italy

## Abstract

**Background:**

To understand the molecular basis of temporomandibular joint (TMJ) pathologies, we aimed to investigate the lubricin levels in the TMJ synovial fluid (SF) of patients with mild to severe internal derangements (IDs).

**Material and Methods:**

A total, 34 joints were the study group. Only patients, with a Wilkes stage of III, IV and V were included, in this sample. Control group consisted of SF from eight joints, from patients undergoing to orthognatic surgery. Concentrations of lubricin in the SF from both samples were measured using ELISA system.

**Results:**

The mean lubricin concentration was 7.029 ± 0.21 µg/mL in stage III patients; 5.64 ± 0.10 µg/mL in stage IV patients, and 4.78 ± 0.11 µg/mL in stage V patients. The lubricin levels from stage IV and stage V patients differed significantly (*P* ≤ 0.001) from those of control subjects. Lubricin levels were inversely correlated with age and to VAS score.

**Conclusions:**

The results of this cross-sectional study highlight the relationship between disease severity and the levels of lubricin in TMJ SF. Our findings suggest that novel biotherapeutic approaches, including the administration of recombinant lubricin in the joint cavity, for the treatment of TMJ diseases can be developed.

**Key words:**Lubricin, TMJ, derangements, synovial fluid.

## Introduction

Temporomandibular disorders (TMDs) are conditions that affect the temporomandibular joint (TMJ) and related musculoskeletal structures. Normally, these disorders are divided into classical myofascial pain, internal derangement (ID), and osteoarthrosis (OA) (1).

Clinically speaking, mild TMJ-ID is characterized by disc displacement, with or without osseous remodeling, whereas severe derangement includes disc or attachment perforations, osseous remodeling, and osteoarthritic changes. OA is a degenerative change in the TMJ that alters its physical and functional properties, the disorder leads to the reversible, and subsequently irreversible, inability of the TMJ to withstand loading stress.

Wilkes proposed the classification, which is commonly used for describing the severity of TMJ diseases ( Table 1) but is also extremely useful for diagnosis (2).

**Table 1:**
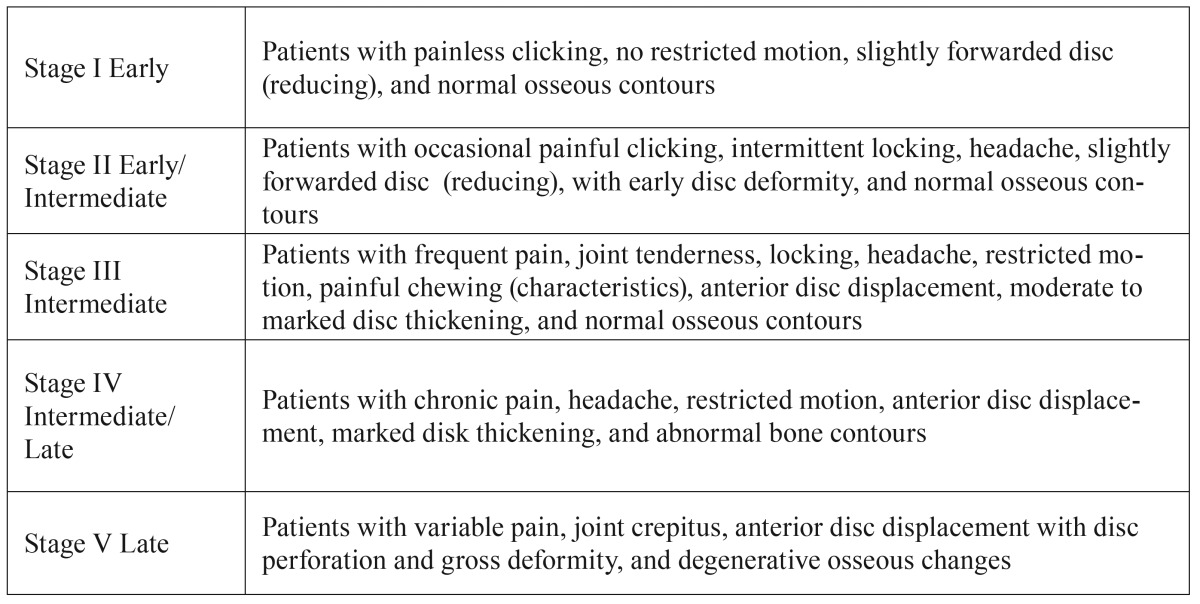
Wilkes classification of TMJ diseases.

Wilkes’s staging is based on the progression of the gross pathology of ID and OA in the joint. The Wilkes classification consists of five stages based on clinical, radiologic and intraoperative findings; it varies from a slight forward displacement with symptom-free normal joints to degenerative arthritic changes with severe clinical symptoms (3). The chronic degenerative tissue changes whereas OA experienced in TMJ disorders may affect articular cartilage and adjacent joint connective tissue responses, including the articular capsule and ligaments, synovial membrane, and the articular bone.

Specific circumstances have been linked to the development of TMJ-OA, including impairment of the synovial fluid (SF) (2,4). Indeed, compromised lubrication in the TMJ is associated with altered frictional properties and surface wear of the condylar cartilage, which is accompanied by the release of pro-inflammatory and matrix degradation mediators under mechanical loading conditions (5). Several molecules that are present in the SF contribute to joint lubrication, particularly boundary lubrication; one such molecule is lubricin (4,6). A longstanding TMJ disc injury affects lubricin expression in the TMJ disc tissue (7). In an investigation of lubricin levels in the SF of patients with TMJ disorders, lubricin was down regulated in patients affected by OA versus patients affected by displaced disc with or without reduction (8). However, any correlation of lubricin levels with Wilkes’s stages, signs and/or symptoms was not provided.

In order to gain a better understanding of the degenerative change mechanism and the molecular basis of OA etiopathogenesis, we decided to use this study to investigate the lubricin levels in the TMJ SF of patients with mild to severe ID. The observed lubricin concentrations were, therefore, correlated with the Wilkes stages, patient ages, visual analog scale (VAS) scores, and maximum interincisal mouth (MIO) opening measurements.

## Material and Methods

- Subjects

The study subjects were selected from a consecutive series of patients who attended the Orofacial Pain and TMD Clinic in the Department of Oral and Maxillofacial Surgery at the Pontifical Catholic University of Paraná, Brazil, from February 2009 to May 2013.

This cross-sectional study was approved by the Ethical Committee on Research at Pontifical Catholic University of Paraná, according to Resolution 196/96 of the National Health Council, and it was approved under registration number 104. A signed informed consent form was also obtained from each patient before collecting the TMJ SF.

**Figure 1:**
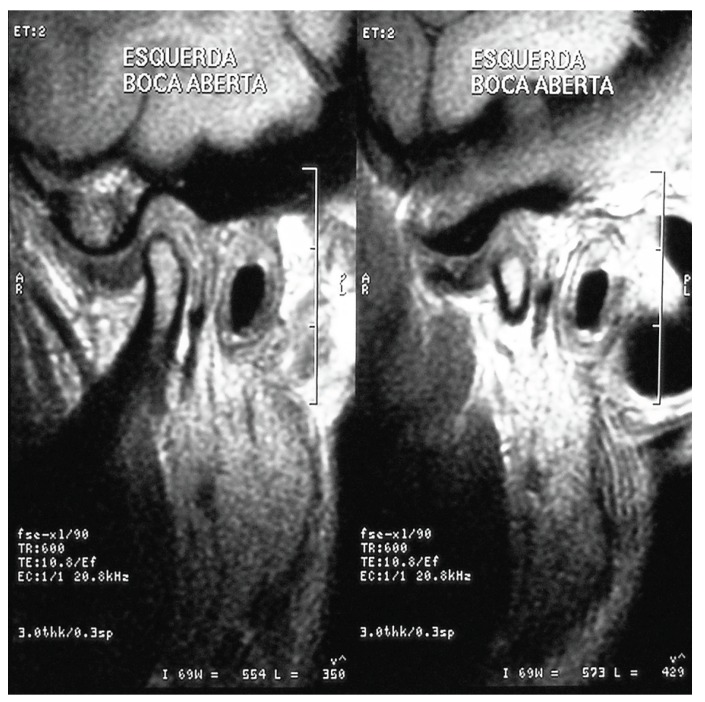
MRI findings of Anterior disc displacement without reduction.

Clinical TMD was diagnosed according to the clinical diagnostic criteria published by Truelove *et al.* (9). All patients were subject to a standard pre-operative examination by the same clinician and were then asked to fill in a VAS to assess pain with endpoint scores going from 0 (no pain) to 10 (worst pain ever experienced). The MIO opening was measured with a ruler to the nearest millimeter. Joint sounds were evaluated by using finger palpation and a stethoscope. Joint and muscle tenderness were also assessed by finger palpation. The diagnosis was based on the clinical status but was also supported by the findings from computed tomography, magnetic resonance imaging, or both (Fig. 1). Following these evaluations, the patients were classified according to their appropriate Wilkes stage (2).

The inclusion criteria were a diagnosis of Wilkes stage (2) III, IV, or V. The exclusion criteria were as follows: a diagnosis of Wilkes stage I or II, systemic arthropathy, use of nonsteroidal anti-inflammatory drugs, and/or history of trauma.

A total number of 34 joints from 32 patients (5 males and 27 females; age range: 21-62 years; mean age: 34 ± 11.04) were included in the study group. Two patients included in this study group had bilateral TMJ involvement. The preoperative clinical signs and symptoms of the patients, defined according to the Wilkes stages, are shown in  Table 2. The mean duration of these symptoms was 4 years (range: 0.7-18 years).

**Table 2:**
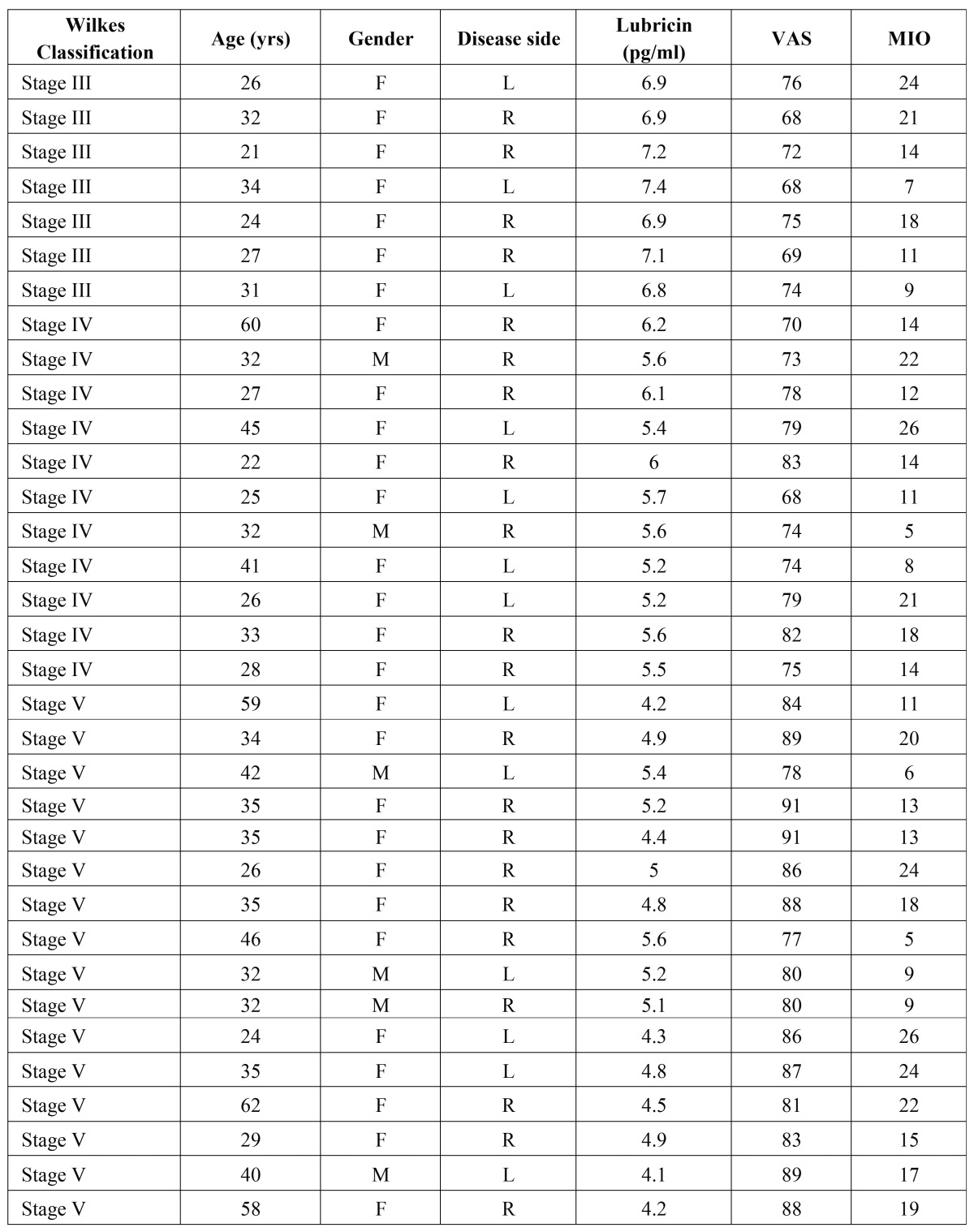
Patient background data. Abbreviations: F: Female; M: Male; MIO: Maximum interincisal opening; visual analog scale (0-100).

The control group consisted of SF from eight joints from eight patients (two males and six females; age range: 28-54 years, (mean age: 42.3 ± 9,5) without signs or symptoms of TMJ disorders. These control patients were volunteers who were undergoing orthognathic surgery and had agreed to participate in the study.

- Collection of the TMJ SF 

Prior to arthrocentesis (under local anesthesia), the SF samples were collected from the superior joint cavity using diluted aspiration as previously described. Briefly, the skin surface was disinfected and SF was aspirated from the affected side of the patients with TMJ disorder and the right-sided TMJ of the control subjects. Physiological saline (1.0 mL) was injected into the upper joint cavity using a 21-gauge hypodermic needle. The SF was collected using the push-and-pull method, which involves repeating the aspiration and injection approximately five times. Afterwards, the collected SF was centrifuged (4°C, 10 min, 1500 ×g) prior to measuring the lubricin concentrations. Only the supernatant was separated and stored in a deep freezer (-80°C). The total amount of protein from each sample was determined by measuring the optical density at 280 nm, with bovine serum albumin used as the standard.

- Enzyme-linked immunosorbent assay (ELISA) analysis of lubricin in the SF

Concentrations of lubricin in the SF were measured using a commercially available ELISA system (Pierce Biotechnology, Rockford, IL, USA) as previously described, (10,11) following the manufacturer’s instructions for the quantitative determination of lubricin. The assay sensitivity of lubricin was 3.25 pg/mL. Concentrations of lubricin less than minimal sensitivity were considered as 0 pg/mL. The level of lubricin was defined as its concentration per milligram of total protein in the SF.

- Statistical analysis

Mean values and standard deviations were calculated for lubricin levels in TMJ SF in each subgroup of the sample (i.e., Wilkes stages III, IV, and V). A Shapiro–Wilk test was used to test for normality. Data had a non-parametric distribution, therefore, Mann–Whitney U tests were used to evaluate any statistical differences among the lubricin levels of the three Wilkes stage subgroups and the control group.

A non-parametric Spearman’s rank correlation test (rho) was used to obtain the correlation coefficient from correlations between lubricin levels and patient ages, VAS scores, and MIO. *P*-values ≤ 0.05 were considered statistically significant. All data were analyzed using the SPSS program (SPSS release 16.0; SPSS, Chicago, IL, USA).

## Results

The distribution of patients according to the Wilkes classification was as follows.

seven patients were stage III, 11 patients were stage IV, and 14 patients were stage V ( Table 2).

The SF lubricin concentration obtained from normal joints was 7.425 ± 0.34 µg/mL. On the contrary, in the study group the lubricin levels progressively decreased as the Wilkes stage became more severe. Specifically, the mean lubricin concentration was 7.029 ± 0.21 µg/mL in stage III patients; 5.64 ± 0.10 µg/mL, in stage IV patients, and 4.78 ± 0.11 µg/mL in stage V patients. The lubricin levels from stage IV and stage V patients differed significantly (*P* ≤ 0.001) from those of control subjects, ( Table 3).

**Table 3:**
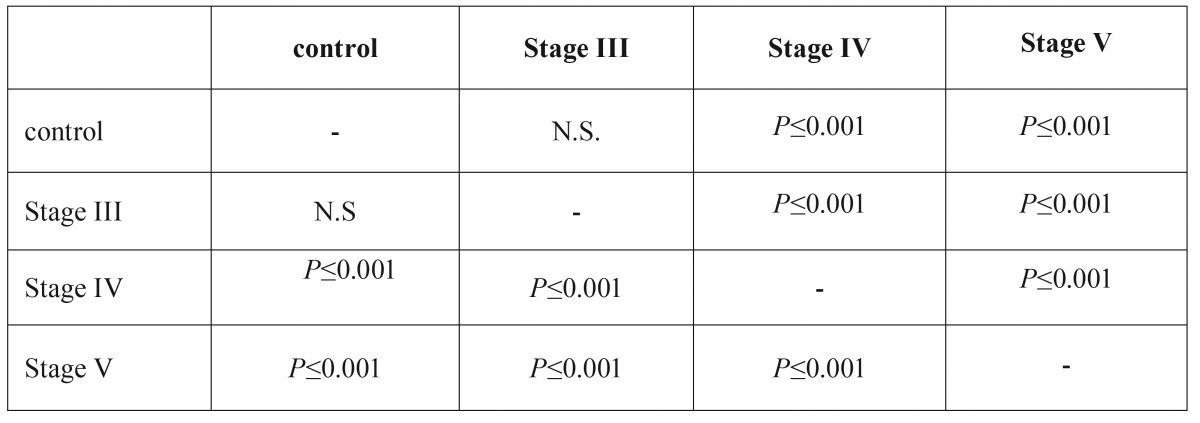
Statistical differences and levels of significance, among the lubricin concentrations in the three Wilkes stage subgroups and the control group according to Mann–Whitney U tests.

**Figure 2:**
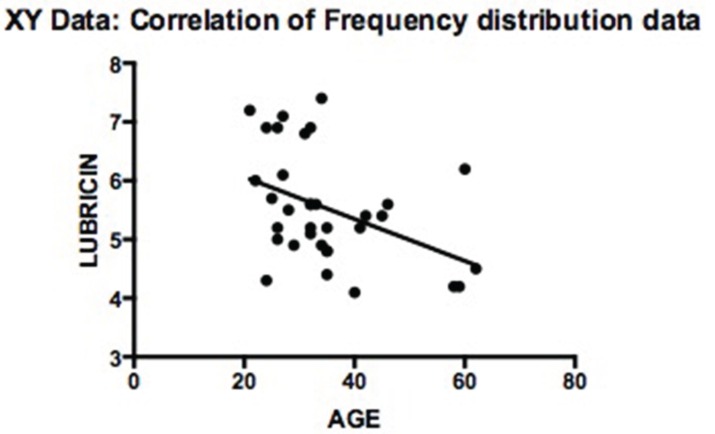
Correlation analysis of lubricin concentrations and patient age.

**Figure 3:**
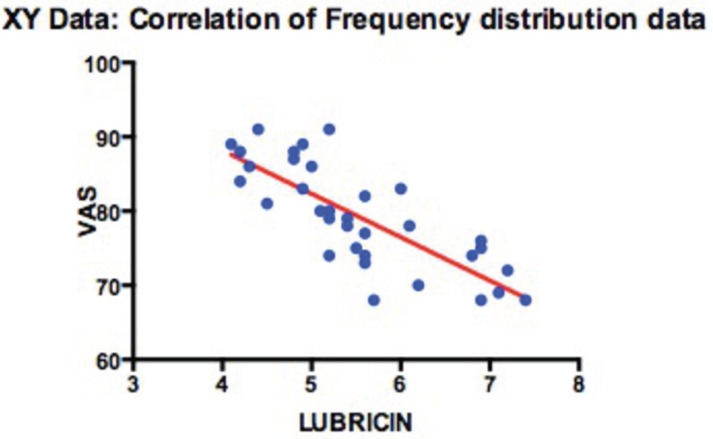
Correlation analysis of lubricin concentrations and VAS.

The lubricin levels were inversely correlated with age (rho = −0.445; *P* ≤ 0.008) (Fig. 2) and to VAS score (rho = −0.823; *P* ≤ 0.009) (Fig. 3); on the other hand, there was no statistically significant correlation between the lubricin levels and MIO.

## Discussion

Articular cartilage functions as a surface that allows joint movement under conditions of extremely low friction. Hyaluronic acid (HA) and lubricin are two major joint lubricants that protect surfaces from wear (12). The mechanically trapped HA–lubricin complex acts as an effective chemically bound lubricant that slightly reduces friction forces but more importantly eliminates wear damage to the cartilage surfaces. Thus, the lubrication system as a whole requires both HA and lubricin to function optimally (11-13). Lubricin is a proteoglycan that is specifically synthesized and expressed by articular chondrocytes of the superficial zone (14).

Lubricin may be degraded and its synthesis may be reduced due to a loss of joint lining cells (the major lubricin producers) or inhibition of lubricin synthesis by inflammatory cytokines. Indeed, numerous cytokines and growth factors are reportedly involved in the regulation of lubricin expression. For example, TGF‑β1, an anabolic factor, up-regulates the synthesis and surface localization of lubricin, whereas IL‑1β or TNF-α down-regulates the production of lubricin (15,16).

It has also been suggested that the altered joint mechanics in knee injuries can lead to decreased lubricin expression in the superficial layer as well as decreased lubricin coating of the surface of articular cartilage. These effects are significant risk factors for the development of secondary OA (17,18). In fact, following a traumatic knee injury, articular cartilage surfaces are predisposed to wear-induced damage because of the resultant changes in joint lubricating mechanisms, which are mediated by lubricin catabolism, and down-regulation due to elevated inflammatory cytokines levels (17). Therefore, lubricin is deficient in arthritic joints (11,19,20).

As far as TMJ is concerned, some studies on animals have highlighted the essential role of lubricin in the maintenance of TMJ integrity and in the earlier onset of TMJ-OA (21,22). However, very few studies have investigated lubricin expression in human TMJ fluid and tissues (7,8). From these few investigations, it appears that lubricin was down-regulated in diseased TMJ disc tissue from patients with IDs (7). Moreover, lubricin concentrations in SF were apparently lower in patients with OA but not in patients with IDs (8).

Findings from our study are in agreement with this previous data. We also demonstrated that the patients’ lubricin levels progressively decreased as their Wilkes stage became more severe and that the lubricin levels were inversely correlated with patient age and VAS (but not with MIO). Our results are corroborated by previous findings obtained in studies from joints other than the TMJ, these studies demonstrated that Lubricin/PRG4 gene expression is affected by aging, which may lead to altered mechanical joint function in elderly subjects (23).

The discovery that lubricin levels are inversely correlated with pain (i.e., with VAS scores) is a novel result in the present study. It could be argued that this latter result could be somehow related to the release of pro-inflammatory and matrix degradation mediators in the SF of patients with TMJ disease.

Regarding this, previous studies reported that in TMJ SF, the TNF-α levels increased in patients with IDs as the stage of the disease progresses, pain levels (VAS scores) increased too. Thus, AA (24) concluded that TNF-α may contribute to the pathogenesis of TMJ cartilage and bone degeneration. In addition, TNF-α induces the production of other pro-inflammatory cytokines such as IL-1, IL-6, and IL-8 (25), which in turn determines the down-regulation of lubricin (17).

Further evidence is provided from another study which demonstrated that under mechanical loading conditions, there is a release of pro-inflammatory and matrix degradation mediators which are associated with altered frictional properties and surface wear of condylar cartilage (5).

Thus, given these previous findings and our present data, it can be hypothesized that in the TMJ, similar to other joints, both altered loading and inflammation can lead to decreased lubricin levels, which in turn play a role in the onset of degenerative changes in joints.

In conclusion, the results of this cross-sectional study highlight the relationship between disease severity and the levels of lubricin in TMJ SF. Significantly, our findings suggest that novel biotherapeutic approaches for the treatment of OA, including the administration of recombinant lubricin in the joint cavity, could be developed in future for the prevention of cartilage degeneration.
